# Supplementary material for: Effects of Different Types of Exercise Training on Pulmonary Arterial Hypertension: A Systematic Review
Source: J Clin Med. 2020 Jun 2;9(6):1689. doi: 10.3390/jcm9061689 (PMC7356848; doi:10.3390/jcm9061689)
Supplement: Supplementary file 1 [file jcm-09-01689-s001.pdf]

Effects of different types of exercise training on pulmonary arterial hypertension: A systematic review

Exercise in PAH

Waller et al.

Supplementary material

Table S1. Overview on the headings and search terms used for this systematic review. Search terms were combined by the Boolean search operator 'AND'.

Headings	Search Terms (Key words)
Exercise training	aerobic exercise training, aerobic training, exercise, exercise training, physical activity, training, exercise training, rehabilitation<br><br>*Related terms and synonyms*<br>anaerobic training, circuit training, strength training, endurance training, chronic training, gymnastics, strengthening, fitness training, calisthenics, sport therapy, cardiovascular training, exercising, physical exercise, physical training, physical motion, motion activity, sporting activity, sport/s activity, workout, movement, weight training, weight bearing exercise
Pulmonary arterial hypertension (PAH)	pulmonary arterial hypertension (PAH), pulmonary hypertension (PH), monocrotaline (MCT)<br><br>*Related terms and synonyms*<br>cardiovascular disease, group 1 pulmonary hypertension (PH), precapillary pulmonary hypertension

Table S2. Detailed search strategy including date, searched databases, and keywords. Electronic databases were searched until July 2019, using medical subject heading terms and related terms. The article type was filtered for randomised and non-randomised controlled trials (RCT & non-RCT), observational studies and clinical trials and only studies in English and German were included.

No.	Title	Author	Year
***Date:* 03.06.2019**; *Database:* Pubmed → Medline, Web of Science; *Key words:* pulmonary arterial hypertension (PAH), exercise training, pulmonary hypertension			
1	Benefits of skeletal-muscle exercise training in pulmonary arterial hypertension: The WHOLEi + 12 trial	González-Saiz, L. et al.	2017
2	Exercise training improves peak oxygen consumption and haemodynamics in patients with severe pulmonary arterial hypertension and inoperable chronic thrombo-embolic pulmonary hypertension: a prospective, randomized, controlled trial.	Ehlken, N. et al.	2016
***Date:* 04.06.2019**; *Database:* Pubmed → Medline, Web of Science; *Key words:* aerobic exercise training, pulmonary arterial hypertension (PAH)			
3	Cardioprotective effects of early and late aerobic exercise training in experimental pulmonary arterial hypertension	Moreira-Goncalves, D. et al	2015
4	Effects of aerobic exercise training on metabolism of nitric oxide and endothelin-1 in lung parenchyma of rats with pulmonary arterial hypertension	Zimmer, A. et al.	2017
5	Effect of aerobic exercise training on fatigue and physical activity in patients with pulmonary arterial hypertension	Weinstein, Ali A. et al.	2013
***Date:* 05.06.2019**; *Database:* Pubmed → Medline, Web of Science; *Key words:* exercise training, pulmonary hypertension (PH), pulmonary arterial hypertension (PAH), exercise			
6	The Combination of Exercise and Respiratory Training Improves Respiratory Muscle Function in Pulmonary Hypertension	Kabitz, H.-J. et al.	2013
7	Oscillatory whole-body vibration improves exercise capacity and physical performance in pulmonary arterial hypertension: a randomised clinical study	Gerhardt, F. et al.	2017
***Date:* 07.06.2019**; *Database:* Pubmed → Medline, Web of Science; *Key words:* exercise training, pulmonary hypertension (PH), pulmonary arterial hypertension (PAH), rehabilitation			
8	Exercise and Respiratory Training Improve Exercise Capacity and Quality of Life in Patients With Severe Chronic Pulmonary Hypertension	Mereles, D. et al.	2006
9	Exercise training in pulmonary arterial hypertension associated with connective tissue diseases.	Grünig, E. et al.	2012
10	Magnetic resonance imaging to assess the effect of exercise training on pulmonary perfusion and blood flow in patients with pulmonary hypertension	Ley, S. et al.	2013
11	Benefits of Intensive Treadmill Exercise Training on Cardiorespiratory Function and Quality of Life in Patients With Pulmonary	Chan, L. et al.	2013

No.	Title	Author	Year
	Hypertension		
12	Effects of an Outpatient Service Rehabilitation Programme in Patients Affected by Pulmonary Arterial Hypertension: An Observational Study	Bussotti, M. et al.	2017
Date: **08.06.2019**; *Database:* Pubmed → Medline, Web of Science*; Key words:* exercise training, pulmonary hypertension (PH), pulmonary arterial hypertension (PAH), rehabilitation			
13	Safety and efficacy of exercise training in various forms of pulmonary hypertension	Grünig, E. et al.	2012
14	Effect of Exercise and Respiratory Training on Clinical Progression and Survival in Patients with Severe Chronic Pulmonary Hypertension	Grünig, E. et al.	2011
15	Efficacy of exercise training in pulmonary arterial hypertension associated with congenital heart disease	Becker-Grünig, T. et al.	2013
16	Effects of exercise training in patients with idiopathic pulmonary arterial hypertension	de Man, F. S. et al.	2009
17	Ambulatory rehabilitation improves exercise capacity in patients with pulmonary hypertension	Fox, B. et al	2011
Date: **10.06.2019**; *Database:* Pubmed → Medline, Web of Science*, Key words:* exercise training, pulmonary arterial hypertension (PAH), pulmonary hypertension (PH), training			
18	Exercise Training in Patients with Pulmonary Arterial Hypertension: A Case Report	Shoemaker, M. J. et al.	2009
19	A prescribed walking regimen plus arginine supplementation improves function and quality of life for patients with pulmonary arterial hypertension: a pilot study	Brown, M. B. et al.	2018
20	High-intensity interval training, but not continuous training, reverses right ventricular hypertrophy and dysfunction in a rat model of pulmonary hypertension	Brown, M. B. et al.	2017
Date: **12.06.2019**; *Database:* Pubmed → Medline, Web of Science*; Key words:* pulmonary hypertension (PH), pulmonary arterial hypertension (PAH), aerobic training, monocrotaline (MCT), rehabilitation, exercise			
21	Rehabilitation Program in Adult Congenital Heart Disease Patients with Pulmonary Hypertension	Martínez-Quintana, E. et al.	2010
22	Voluntary exercise delays heart failure onset in rats with pulmonary arterial hypertension	Natali, A. et al.	2015
23	Exercise preconditioning prevents MCT-induced right ventricle remodeling through the regulation of TNF superfamily cytokines	Nogueira-Ferreira, R. et al.	2016
24	Preventive aerobic training exerts a cardioprotective effect on rats treated with monocrotaline	Pacagnelli, F. L. et al.	2016
Date: **13.06.2019**; *Database:* Pubmed → Medline, Web of Science; *Key words:* exercise training, monocrotaline (MCT), aerobic exercise, pulmonary hypertension, exercise			
25	Effects of a chronic exercise training protocol on oxidative stress and right ventricular hypertrophy in monocrotaline-treated	Souza-Rabbo, M. P.	2008

No.	Title	Author	Year
	rats	et al.	
26	Effects of exercise on monocrotaline-induced changes in right heart function and pulmonary artery remodeling in rats	Colombo, R. et al.	2013
27	Aerobic Exercise Promotes a Decrease in Right Ventricle Apoptotic Proteins in Experimental Cor Pulmonale	Colombo, R. et al.	2015
28	Exercise training contributes to H2O2/VEGF signaling in the lung of rats with monocrotaline-induced pulmonary hypertension	Colombo, R. et al.	2016
Date: **15.06.2019**; *Database*: Pubmed → Medline, Web of Science; *Key words*: pulmonary hypertension, exercise training, pulmonary arterial hypertension (PAH), training			
29	Opposite Effects of Training in Rats With Stable and Progressive Pulmonary Hypertension	Handoko, M. L. et al.	2009
30	Downhill exercise training in monocrotaline-injected rats: Effects on echocardiographic and haemodynamic variables and survival	Enachea, I. et al.	2017
31	Short term effects of exercise training on exercise capacity and quality of life in individuals with pulmonary arterial hypertension	Ganderton et al.	2012
Date: **10.07.2019**; *Database:* Pubmed → Medline, Web of Science, UB; *Key words:* pulmonary arterial hypertension (PAH), rehabilitation			
32	Effects of a Rehabilitation Program on Skeletal Muscle Function in Idiopathic Pulmonary Arterial Hypertension	Mainguy, V. et al.	2010

Table S3. Functional classification of patients in human studies. WHO: World Health Organisation; NYHA: New York Heart Association.

Author (year)	Patients	WHO & NYHA Functional classification			
	(n)	I	II	III	IV
[11] Mereles et al. (2006)	30	0	6	22	2
[88] Man et al. (2009)	19	0	3	16	0
[76] Shoemaker et al. (2009)	2	0	2	0	0
[89] Mainguy et al. (2010)	5	0	3	2	0
[90] Martínez-Quintana et al. (2010)	8	0	3	5	0
[91] Fox et al. (2011)	22	0	13	9	0
[37] Grünig et al. (2011)	58	0	10	44	4
[92] Ganderton (2012)	10	0	6	4	0
[12] Grünig et al. (2012a)	183	2	26	137	18
[93] Grünig et al. (2012b)	21	0	9	7	5
[36] Becker-Grünig et al. (2013)	20	0	6	14	0
[69] Chan et al. (2013)	23	1	12	9	1
[94] Ley et al. (2013)	20	0	4	16	0
[87] Weinstein et al. (2013)	24	1	12	10	1
[95] Kabitz et al. (2014)	7	0	0	6	1
[96] Ehlken et al. (2016)	87*	0	14	66	4
[97] Bussotti et al. (2017)	15	0	9	6	0
[73] Gerhardt et al. (2017)	22	0	13	9	0
[98] González-Saiz et al. (2017)	40	9 (+4 in I-II)	21 (+4 in II-III)	2	0
[77] Brown et al. (2018)	12	0	6	6	0

*missing values for three patients

Table S4. Detailed list of reporting of adverse events. All 20 human studies mentioned in this review article were screened for addressing of study-related side effects.

Author (year)	Reported adverse events
[11] Mereles et al. (2006)	None
[37] Grünig et al. (2011)	None
[69] Chan et al. (2013)	None
[76] Shoemaker et al. (2009)	None
[77] Brown et al. (2018)	None
[88] Man et al. (2009)	None
[90] Martínez-Quintana et al. (2010)	None
[91] Fox et al. (2011)	None
[92] Ganderton et al. (2012)	None
[97] Bussotti et al. (2017)	None
[87] Weinstein et al. (2013)	No information provided
[89] Mainguy et al. (2010)	No information provided
[94] Ley et al. (2013)	No information provided
[95] Kabitz et al. (2014)	No information provided
[96] Ehlken et al. (2016)	No information provided
[98] González-Saiz et al. (2017)	No information provided
[36] Becker-Grünig et al. (2013)	Respiratory infections
[12] Grünig et al. (2012a)	Respiratory infections, (pre-)syncope
[73] Gerhardt et al. (2017)	Back pain, sore muscles
[93] Grünig et al. (2012b)	Gastrointestinal infections with diarrhea, respiratory infections
